# A systematic review of the physical activity levels of acutely ill older adults in Hospital At Home settings: an under-researched field

**DOI:** 10.1007/s41999-020-00414-y

**Published:** 2020-10-15

**Authors:** Jennifer Scott, Ukachukwu O. Abaraogu, Graham Ellis, Maria Giné-Garriga, Dawn A. Skelton

**Affiliations:** 1 Centre for Living, School of Health and Life Sciences, Glasgow Caledonian University, Glasgow, UK; 2 NHS Lanarkshire, Monklands Hospital, Monkscourt Ave, Airdrie, UK; 3 Blanquerna Faculty of Psychology, Education and Sport Sciences, Ramon Llull University, Barcelona, Spain; 4 Blanquerna Faculty of Health Sciences, Ramon Llull University, Barcelona, Spain; 5 Department of Medical Rehabilitation, University of Nigeria, Enugu, Nigeria

**Keywords:** Aged, Systematic review, Hospital at home, Physical activity, Accelerometry

## Abstract

**Purpose:**

The purpose of this review was to identify, evaluate and synthesise existing evidence reporting the physical activity levels of acutely ill older patients in a ‘Hospital At Home’ setting and compare this to patients with similar characteristics treated in a traditional hospital inpatient setting. Functional changes and any adverse outcomes due to physical activity (e.g. falls) in both settings where PA was reported or recorded were also evaluated as secondary outcomes.

**Methods:**

A search strategy was devised for the MEDLINE, CINAHL, AMed, PEDRO, OT Seeker and Cochrane databases. Search results were title, abstract and full-text reviewed by two independent researchers. Data were extracted from included articles using a custom form and assessed for quality and risk of bias using the Appraisal Tool for Cross-Sectional Studies.

**Results:**

No studies set in the Hospital at Home environments were identified. 16 hospital inpatient studies met the criteria for inclusion. Older patients managed in inpatient settings that would be eligible for Hospital at Home services spent 6.6% of their day active and undertook only 881.8 daily steps. Functional change was reported in four studies with both improvement and decline during admission reported.

**Conclusion:**

There is a lack of published research on the physical activity levels of acutely-ill older adults in Hospital at Home settings. This review has identified a baseline level of activity for older acutely ill patients that would be suitable for Hospital at Home treatment. This data could be used as a basis of comparison in future hospital at home studies, which should also include functional change outcomes to further explore the relationship between physical inactivity and functional decline.

**Electronic supplementary material:**

The online version of this article (10.1007/s41999-020-00414-y) contains supplementary material, which is available to authorized users.

## Key summary points

**Aim:**

To identify, evaluate and synthesise the evidence concerning the physical activity levels of acutely-ill older patients undergoing ‘Hospital At Home’ treatment compared to those of patients with similar characteristics in a traditional hospital inpatient setting.

**Findings:**

No studies on the physical activity levels of acutely ill older adults in Hospital At Home Settings were identified. Patients managed in inpatient settings that would be eligible for Hospital At Home services spend 6.6% of their day active and perform only 881.8 daily steps, placing them at increased risk of functional decline.

**Message:**

There is a lack of published research on physical activity in acutely ill older adults in Hospital At Home sttings; further research is needed.

**Electronic supplementary material:**

The online version of this article (10.1007/s41999-020-00414-y) contains supplementary material, which is available to authorized users.

## Introduction

Hospital at Home (HaH) is a model of healthcare delivery which provides an alternative to hospitalisation by delivering acute-level hospital services in a residential setting [1]. The HaH care model has increased in prevalence in recent years, with well-established programmes providing services in Western Europe, North America, Brazil, Australia, Israel and South East Asia [2]. Home-hospitalisation has also been advocated during the recent COVID-19 pandemic as a means of increasing bed capacity, facilitating quarantine and reducing disease transmission to vulnerable groups [3]. Research interest has also been growing, with a more than sixfold increase in HaH-related citations between 1999 and 2019 [4]. A recent systematic review found that HaH may be a clinically effective alternative to inpatient care for some older, acutely-ill medical patients [5]. Furthermore, it suggested HaH treatment may pose less risk of physical functional decline to patients than the traditional ward-based inpatient environment [5]. Functional decline is a known adverse effect of hospitalisation, affecting between 30 and 56% of older inpatients between admission to hospital and discharge [6–9], manifesting as a loss of muscle mass, strength, physical function and/or ability to perform basic activities of daily living such as dressing, eating and maintaining hygiene and continence [10–12].

Physical inactivity while hospitalised, combined with older age, are predictors of functional decline [13]. Hospitalised patients are highly inactive, with acute medical and surgical inpatients spending between 93 and 98.8% of their time sitting or lying [14], and older patients spending as little as 76mins per day in an upright position [15]. Recently published draft recommendations on physical activity for inpatients have emphasised the importance of incorporating opportunities for physical activity into the daily care of older adults to improve clinical outcomes, focusing on function, independence and activities of daily living [16]. However, there are many institutional barriers to physical activity in hospital including lack of staff support, tethering to medical devices, lack of assistive devices, and unfamiliar surroundings, as well as a fear of injury [17]. Treatment in a less restrictive home environment may overcome such barriers, providing more opportunity for patients to continue to perform regular activities of daily living [5], thereby lessening the risk of functional decline.

This review sought to investigate the hypothesis that older, acutely ill patients treated in a HaH setting may be more active than hospital inpatients with similar characteristics. The aim was to identify, evaluate and synthesise primary research studies reporting cumulative physical activity levels in these populations and, where reported, evaluate reports of functional decline or adverse effects resulting from physical activity during admission. As will be reported, no studies conducted in HaH treatment settings were identified, and functional change outcomes were largely absent.

## Methods

The review protocol was developed in accordance with Preferred Reporting Items for Systematic review and Meta-Analysis Protocols (PRISMA-P) [18] guidelines and registered with the International Prospective Register of Systematic Reviews (PROSPERO, Registration Number CRD42019138822) [19]. The review followed the guidelines set out in the Cochrane Handbook for Systematic Reviews of Interventions [20] where applicable and complies with the PRISMA Statement [21] for the conduct and reporting of systematic reviews.

### Search strategy

A comprehensive search strategy was developed in accordance with the Cochrane Recommendations for Health Care Review [22] and reviewed by a specialist medical librarian. The search was initiated in July 2019 and updated 19 January 2020 to ensure currency. Search terms and appropriate synonyms were chosen in alignment with the research objective and combined using Boolean operators, subject headings, truncations and wildcards where appropriate. Filters limited results to peer reviewed, English language, human studies with available abstracts published since 1980. All study designs were acceptable. The databases MEDLINE (Ovid Interface), CENTRAL, Cumulative Index to Nursing and Allied Health Literature (CINAHL), Allied and Complementary Medicine Database (AMED), PEDro and OTseeker were chosen as the most relevant to the subject matter. The full search strategies with database-specific syntaxes for all sources are included in Online Resource 1. Once key papers were identified, reference lists were hand-searched and subject experts were approached to identify any further resources. ‘Grey’ literature including conference abstracts, reports, unpublished data and dissertations were not included. Multiple publications using the same participant dataset were excluded and the most comprehensive or recent publication used.

### Inclusion/exclusion criteria

*Setting* Studies set in either an HaH or acute medical inpatient environment were included, studies did not have to compare both groups. HaH was defined as ‘a service that provides acute, hospital-level care by healthcare professionals in a home context for a condition that would otherwise require acute hospital inpatient care’ [1]. An acute inpatient setting was defined as ‘a hospital (private or public) providing 24-h care for people who are unwell and had an unplanned admission’ [23]. As HaH is designed to treat acute episodes of transient rather than chronic medical illness [5], studies set in non-medical or non-acute environments such as palliative care, respite, rehabilitation, mental health, long-term care or residential nursing home facilities were excluded. Studies concerned with post-discharge HaH services (e.g. ‘step-down’ HaH), were also excluded, as the focus of the research project is HaH as an alternative to hospital admission for the preservation of physical function.

*Participants* Studies involving patients aged 60 and over diagnosed with an acute-onset medical condition that would fall within the scope of a HaH service were included. HaH services predominantly manage non-surgical, non-critical conditions such as infection, acute exacerbations of cardiac and respiratory conditions, haematological and metabolic disturbances, and acute kidney injury [1]. Certain conditions are not appropriate for management in a home setting such as those requiring surgery (e.g. acute coronary syndromes, orthopaedics), critical care or advanced diagnostics and interventions (e.g. stroke). To ensure that intervention and comparison populations were similar, studies containing these large numbers of patients with such conditions were excluded unless these participants could be discounted from the results. A margin of $\le$ 10% of patients under 60 and $\le$ 10% with excluded conditions was allowed. Where numbers exceeded this margin, or other pertinent information was required, study authors were approached via email on up to 2 occasions to request abridged results. Where a custom dataset was provided, this was used in analysis over the published dataset.

*Intervention and comparator* The intervention of interest was treatment in a HaH setting compared to standard inpatient acute care. As this review aimed to establish if there are differences in the cumulative activity levels of patients in each setting, trials of other interventions to increase patient activity such as exercise programmes or physiotherapy sessions over and above usual care were not suitable for inclusion unless the physical activity levels of the control group were available, as the intervention group would not be representative of the general older acute population.

*Outcome* The primary outcome measure was the cumulative level of PA performed by patients receiving standard medical care in a HaH and/or inpatient setting. It was decided a priori that acceptable measures would include objective methods, such as activity monitor data, or subjective methods, such as direct observation, self-reported instruments or questionnaires.

Changes in functional independence (e.g. Activities of Daily Living, dependent walking) and physical performance (e.g. handgrip test, timed up and go) from admission to discharge, as well as any adverse effects reported as a consequence of physical activity (e.g. falls) were selected as secondary outcomes.

The inclusion and exclusion criteria are summarised in Table 1.

**Table 1:** Inclusion and exclusion criteria

	Inclusion criteria	Exclusion criteria
Setting	Acute medical inpatient or HAH environment	Post-discharge/step-down HAH Pre/post-surgical wards Palliative/end of life care Respite, rehabilitation or recuperation wards Long term care/residential care Mental health admissions
Population	$\ge$ 90% Aged 60 and over Diagnosed with an acute-onset medical condition falling within the scope of an HAH Service	Over 10% of patients admitted for conditions that would not be managed within a HAH setting such as stroke, acute coronary syndromes, surgical or orthopaedic emergencies
Outcome measures	Objectively/subjectively measures amount of physical activity performed by patients while admitted	

### Selection process

**Fig. 1:**
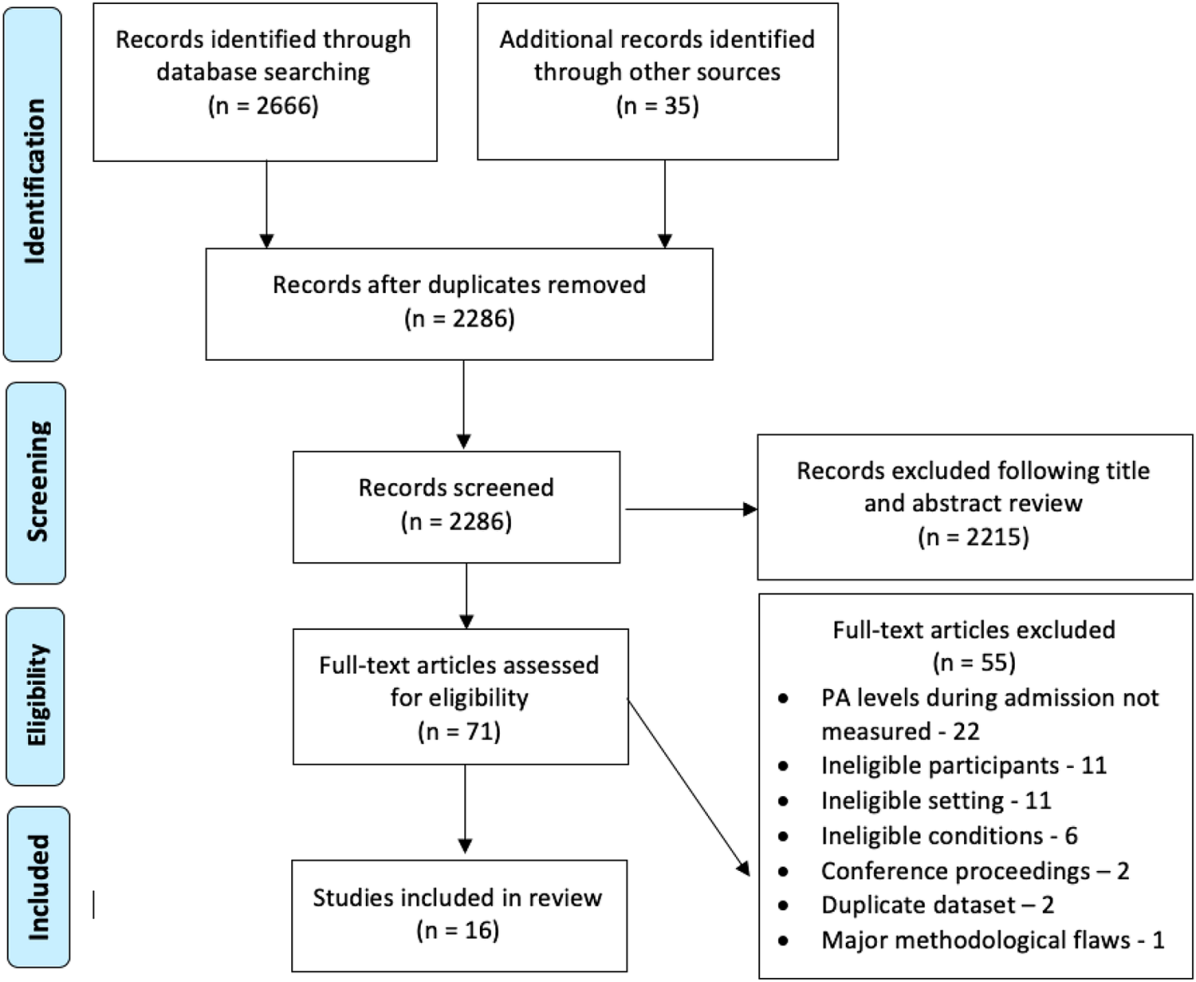
PRISMA flow diagram [21]

Literature search results and bibliographic records were exported into RefWorks to facilitate deduplication and screening of titles and abstracts. Articles meeting the inclusion criteria were then subjected to full-text appraisal. All records were reviewed by the lead researcher (JS) and independently second-reviewed by another (DS, UA, MG or GE). The decision for inclusion or exclusion was recorded along with reasons for exclusion. Where there was disagreement between reviewers on inclusion at any stage, a third reviewer was consulted. Sixteen articles were selected for inclusion in the review. This process for identifying these is documented in the PRISMA flowchart [21] below (Fig. 1).

### Data extraction and analysis

The process of data extraction was performed using a custom template which was developed and piloted to extract: (1) data relevant to the research question, and (2) data required to perform a quality appraisal and risk of bias assessment using the Appraisal Tool for Cross-Sectional Studies (AXIS) [24] (Data Extraction Table: Online Resource 2, AXIS Appraisal: Online Resource 3). The AXIS tool comprises 20 questions and considers study design and reporting quality in addition to the risk of bias when appraising research studies [25]. The data extracted were spot-checked for accuracy by the review team (DS, UA, MG or GE). Where studies reported results for participants that were excluded from this review (e.g. Surgical, non-geriatric) these were separated and excluded from the analysis. Separate datasets were requested and received from Karlsen [26] and Valkenet [27] containing only participants that met the inclusion criteria.

The physical activity outcomes of the studies were grouped according to their method of measuring physical activity levels and reporting format. In accordance with Duvivier [28], standing and slow walking have both been categorised as physical activity and grouped together into ‘active time’ for the purposes of analysis. Time spent sitting or lying down, including sleep time, has been classified as ‘non-active’ time. This classification allowed 3 categories to emerge; (1) Active time recorded over 24 h, (2) Active time recorded over variable timeframes, and (3) physical activity as step count.

The percentage of time spent actively was selected as a common scale to enable comparison of data across the studies. Studies using step count as a measure of physical activity were reported separately. Results reported in minutes were converted into a percentage of 24 h. Median and interquartile ranges were converted into mean values using the formula devised by Wan [29] to allow results to be summarised as pooled averages. Summary independent t-tests were used to examine whether physical activity or step count differed significantly from the pooled averages when grouped by medical condition or studies at lower risk of bias. Analyses were performed using SPSS v26, *p* < 0.05 was considered significant and 95% confidence intervals are reported.

## Results

### Characteristics of included studies

*Study characteristics* No suitable HaH studies were identified. All 16 included studies were conducted in single-site acute inpatient hospital environments. The studies were published between 2006 and 2019, and the majority (*n* = 13) were cross-sectional observational designs aiming to establish the physical activity levels of patients as a primary outcome. This design is consistent with the nature of the research question, which does not aim to evaluate the efficacy of an intervention. Of the remaining three studies, two were Validation/Agreement studies [27, 30], and one was a Randomised Controlled Trial (RCT) [31].

*Participants* Most studies concerned general acute medical patients (*n* = 11, mean sample size 114, range 16–287). Five studies were exclusively concerned with patients with specific conditions; two each reported physical activity levels of patients with acute exacerbations of chronic obstructive pulmonary disease (mean sample size 13.5, range 10–17) [30, 32], and heart failure (mean sample size 36, range 27–45) [33, 35] and whilst one reported on patients with mixed medical conditions plus mild-moderate cognitive impairment (sample size 20) [34].

*Primary outcome* All included studies assessed physical activity levels using objective accelerometer-based methods, except Belala [34] who used behavioural mapping. Valkenet [27] also performed behavioural mapping in addition to accelerometery (Dynaport MoveMonitor). A variety of monitoring devices and algorithms were used, with the ActivPAL (PAL Technologies, Glasgow, UK) being the most commonly used device in studies concerned with posture (5 uses), and the Stepwatch Activity Monitor (Modus health, Washington, US) used most frequently for step count (4 uses). The validity of the methods used was reported by most studies, except for the Mediwalk Pedometer (Terumo, Japan), used by Ueda [31]. The range and validity of outcome measures used is available in Online Resource 4.

### Risk of bias in included studies

The included studies were assessed for risk of bias using the AXIS tool [24] (Online Resource 3) which was deemed appropriate due to the high proportion of observational studies identified. There is an inherent risk of bias in descriptive, observational study designs, which rank low on evidence hierarchies, however, a well-designed and conducted cross-sectional study can be of some evidential value [35]. The AXIS tool prompts consideration of selection, instrumentation and reporting bias as well as reporting and study design quality. It was also suitable for the evaluation of the methodology used to acquire and report physical activity levels in the RCT included in this review [31].

**Fig. 2:**
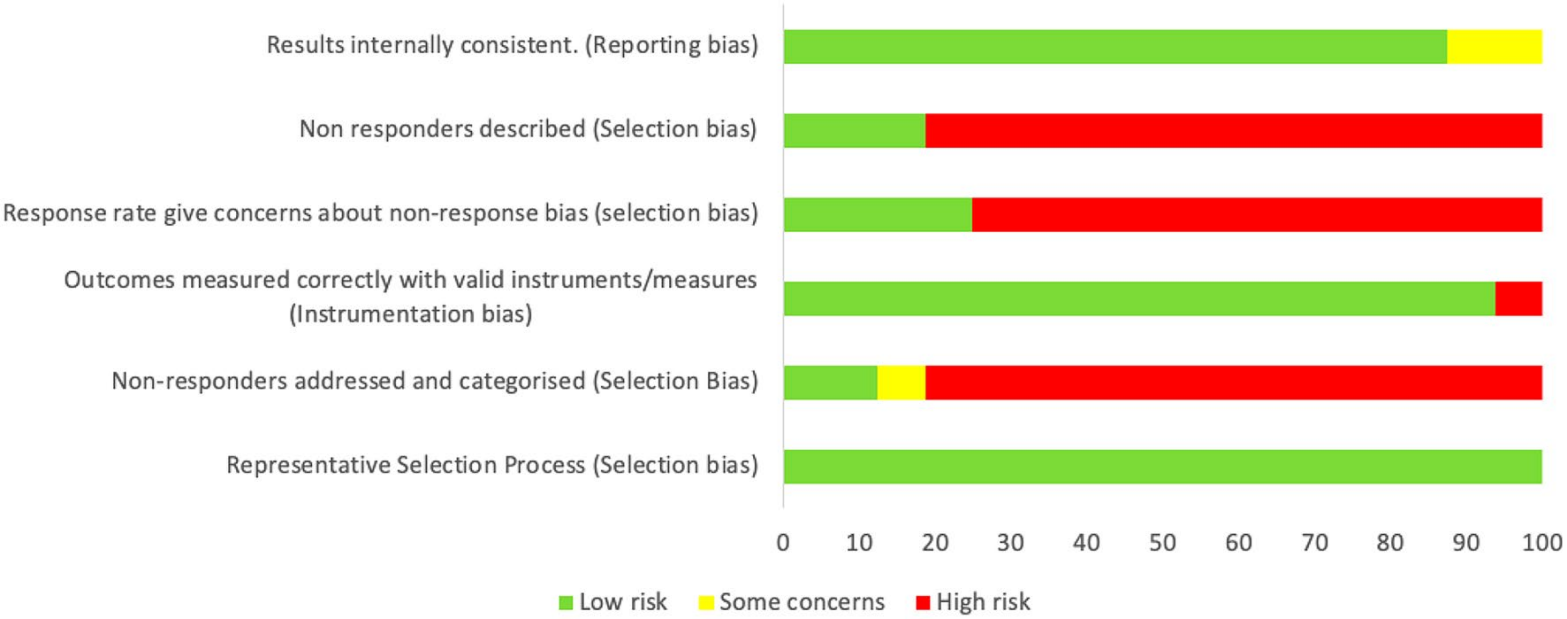
Domain-based risk of bias assessment across all studies

A domain-based risk of bias assessment indicates a low risk of instrumentation and reporting bias, with adequate measurement and reporting of physical activity levels, however, there is a high risk of selection bias within the identified research (Fig. 2). The studies that performed better in the analysis [34, 36–38] gave greater consideration to reporting information on non-responders (patients that were eligible for inclusion but declined to participate).

In terms of quality assessment, overall reporting quality was high, however, study design considerations were less well evidenced, with a broad lack of consideration of sample size, and frequently vague reporting of ethics or consent protocols.

### Physical activity

*Active time recorded over 24 h* The level of inpatient physical activity reported as a percentage of 24 h could be established in seven studies (Table 2). When averages were pooled, the mean proportion of time spent active was found to be 6.6% ± 6.3 (range 3.8–8.3%).

**Table 2:** Active time recorded over 24 h

Study (location)	Participant information	Length of stay (days), disease characteristics	Device, duration of monitoring	Time spent in activity—% of 24 h	Comments/transformations performed
Evenson et al. 2017 [38] (Norway)	*N* = 38 Age: 82.9 ± 6.3 Male:74%	LOS: 11.1 ± 7.8 Diagnoses: mixed acute	ActivPAL (PAL Technologies, Glasgow, UK) Recorded for 24 h on day 3 of admission	8.1% ± 6.2	All hours converted to minutes. All minutes converted to a % of 24 h
Floegel et al. 2019 [35] (USA)	*N* = 27 Age: 78.0 ± 9.8 Male:48%	LOS: 5 ± 3.9 Diagnosis: heart failure	ActivPAL Recorded 24 h a day from recruitment to discharge	7.3% ± 9.6	Results for standing time (5.30% ± 4.2) and ambulating time (1.97% ± 8.6) summed with a pooled SD
Karlsen et al. 2017 [28] (Denmark)	*N* = 151 Age: 85 ± 7.2 Male:35%	LOS: 12.0 ± 6.0 Diagnoses: mixed acute	ActivPAL Recorded 24 h/day from day 2 until discharge or day 13, whichever sooner	8.3% ± 6.6	Authors provided dataset with results for excluded participant groups removed—analysis performed on raw data provided
Rowlands et al. 2014 [32] (Australia)	*N* = 10 Age: 75.9 ± 9.7 Male:40%	LOS: NR Diagnosis: acute exacerbation of COPD	ActivPAL Recorded 24 h/day from day 2 of admission for 1–2 days	7.7% ± 5.6	All hours converted to minutes. All minutes converted to a % of 24 h
Villumsen et al. 2015 [41] (Denmark)	*N* = 100 Age: 84 ± 6.3 Male:40%	LOS: 14.2 ± 8.3 Diagnoses: mixed acute	ActivPAL Recorded 24 h/day from day 3 to discharge	5.5% ± 6	Median results converted to mean. Minutes converted to a % of 24 h
Brown et al. 2009 [42] (USA)	*N* = 45 Age: 74.0 ± 6.5 Male:100%	LOS: 5.2 ± 5.3 Diagnoses: mixed acute	Augmentech Recorded for 7 days or until discharge, whichever sooner	3.8% ± 3.5	No transformation required
Pedersen et al. 2012 [43] (Denmark)	*N* = 42 Age: 83.5 ± 6.6 Male: 55%	LOS: 7.7 ± 1.5 Diagnoses: mixed acute	Augmentech Applied within 48 h of admission, recorded 24 h/day for 10 days or discharge, whichever sooner	4.5% ± 3.3	Median results converted to mean. All hours converted to minutes. All minutes converted to a % of 24 h. 4.2% of time unaccounted for in original results. Non-ambulatory patients in study as immobility reference excluded from this review

All figures mean ± standard deviation (SD) unless stated. *N* number of participants, *LOS* length of stay

*Active time recorded over a variable timeframe* Three studies collected results over shorter, variable timeframes (7–12 h periods), during waking hours, and with different populations and measurement techniques (accelerometery and behavioural mapping), which precludes pooling of results, however, it can be seen that daytime-only levels are higher than the mean for 24 h results, ranging from 8.8 to 13.9% (Median 10.7%) (Table 3).

**Table 3:** Active time recorded over variable timeframe

Study	Participant information	Length of stay (days), disease characteristics	Method/device, duration of recording	Time spent in activity – variable timeframe	Comments/transformations performed
Belala et al. 2019 [36] (Germany)	Number (*N*): 20 Age: 84 ± 6.8 Male: 40%	LOS: 16.9 ± 16.9 Diagnoses: Mixed acute + mild-moderate cognitive impairment	Behavioural Mapping Duration: 10 h (0900–1900), excl. 2 × 45 min breaks Observations: 1 min every 15 mins	13.9%	Inactive time was provided—active time has been extrapolated. SD not available
Pitta et al. 2006 [34] (Belgium)	*N*: 17 Age: 69 ± 14.55 Male: 94%	LOS: 10 days according to local COPD protocol, 3 stayed longer for medical reasons Diagnosis: Acute exacerbation of COPD	Dynaport MoveMonitor Duration: 12 h (0830–2030). Data collected on day 2 and 7 of admission	10.7% ± 11.6	Median results converted to mean. Data collected on the 2nd and 7th. Day of admission only—these results have been averaged
Valkenet et al. 2019 [29] (Holland)	*N*: 16 Age: 72.1 ± 10.3 Male: 63%	LOS: 12.9 ± 7.4 Diagnoses: Mixed acute	Dynaport MoveMonitor Duration: 7 h (0900–1600)	8.8% ± 5	Authors provided dataset with results for excluded participant groups removed. Analysis has been performed on raw data provided

All figures mean ± standard deviation (SD) unless stated. *N* number of participants, *LOS* length of stay

*Physical activity as step count* Eight studies used pedometers or accelerometers to record 24 h step count as a measure of physical activity (Table 4). The pooled mean was 881.8 (1068.2) (range 259.8—1447) steps/24 h.

**Table 4:** Daily Step count

Study	Participant information	Length of stay (LOS) (days), disease characteristics	Device, duration of recording	Mean steps	Comments/transformations performed
Villumsen et al. 2015 [41] (Denmark)	*N*:100 Age: 84 ± 6.3 Male:40%	LOS: 14.2 ± 8.3 Diagnoses: mixed acute	ActivPAL Recorded 24hrs/day from day 3 to discharge	Daily steps: 554 ± 836.5	Median results converted to mean
Ueda et al. 2016 [33] (Japan)	*N*: 45 Age: 82.4 (Whole group) Male: 49% (Whole group)	LOS: 18.1 (Whole group) Diagnoses: heart failure	Mediwalk Pedometer recorded day 2 of admission until day 10	Daily steps: 259.8 ± 389.5	Median results converted to mean. Oral and IV group results for mean age, LOS and steps were combined. Pedometer data available for 45/50 participants (age, gender and LOS are whole group results)
Fisher et al. 2016 [44] (USA)	*N*: 164 Age: 76.3 ± 7 Male: 29%	LOS: 5.2 ± 4.3 Diagnoses: mixed acute	StepWatch Activity Monitor (SAM) Recording 24 h/day from admission to discharge	Steps/24 h: 765 ± 850.4	Median results converted to mean
Lim et al. 2018 [45] (UK)	*N*: 38 Age: 87.8 ± 4.8 Male: 47%	LOS: Mean no. of days recorded: M 4.8. F 3.8 Diagnoses: mixed acute	SAM Recorded 24 h/day for 7 days or until discharge, whichever sooner	Daily steps: 1162.3 ± 914.5	Median results converted to mean. Lower limb accelerometery data only extracted
McCullagh et al. 2016 [39] (Ireland)	*N*: 154 Age: 77.5 ± 7.4 Male: 50%	LOS: 8.1 ± 5.4 Diagnoses: mixed acute	SAM Recorded continuously for first 7 days or until discharge, whichever sooner	Daily steps: 764.4 ± 706	No transformation required
Ostir et al. 2013 [40] (USA)	*N*: 224 Age: All 65 + Male:34%	LOS: Whole group mean not reported Diagnoses: mixed acute	SAM Applied within 24 h of admission, recorded until discharge	Steps/24 h: 883.5 ± 1289.7	Median results converted to mean. Data was collected on the 1st and last 24 h of admission only—these results have been averaged
Floegel et al. 2019 [35] (USA)	*N*: 27 Age: 78.0 ± 9.8 Male:48%	LOS: 5 ± 3.9 Diagnosis: heart failure	Tractivity Recorded 24 h/day from recruitment to discharge	Steps/24 h: 1447 ± 1184	No transformation required
Sallis et al. 2015 [46] (USA)	*N*: 287 Age: All over 65 Male: 43% (whole group)	LOS: 3.98 ± 3.8 (whole group) Diagnoses: mixed acute	Tractivity Recorded 24 h/day from admission to discharge	Steps/24hrs: 1132 ± 1202.9	Data collected on 1st and last 24hrs of admission only – these results have been averaged. Age, gender and LOS details not available for subset of interest (medical patients 65 +)

All figures mean ± standard deviation (SD) unless stated

*N* number of participants, *LOS* length of stay, *IV* intravenous, *SAM* StepWatch Activity Monitor, *M* male, *F* female

### Secondary outcome measures

Functional change between admission and discharge was reported in 4 studies, the results extracted are summarised in Table 5. As will be discussed, the reported outcomes from these studies were highly heterogenous in terms of tools used, data collection protocols and presentation of data, such that no summative conclusions on of the impact of differing physical activity levels on the incidence of functional decline could be drawn from the data.

**Table 5:** Summary of functional change results

Study ID	Measure and protocol	Functional change
Karlsen et al. 2017 [28] (Denmark)	Changes in function were assessed three times during the stay using the deMorton Mobily Index (DEMMI), 30 s Chair Stand Test (30sCST)and Hand grip strength	Results for subset of patient in this review unavailable. Results for all patients, including participants excluded from this review: DEMMI: Score improved by a mean of + 4.2 between test 1 and 3 30sCST: Score improved by a mean of + 1.2 between test 1 and 3 Handgrip strength unchanged
Pitta et al. 2006 [34] (Belgium)	Quadricep Force (in Newton Metres) was recorded on day 3 and 8	Median Quadricep force declined from Day 3 (98, IQR 79–126) to Day 8 (90, IQR 67–109)
Ueda et al. 2016 [33] (Japan)	Changes in function were assessed at baseline and day 10 of admission using the Barthel Index (BI) and Functional Independence Measure (FIM)	Mean score calculated from average of both cohorts show that both BI and FIM scores declined during admission: BI: Baseline: 92.4 ± 12.9. Day 10: 68.9 ± 29.5 FIM: Baseline: 113.8 ± 13.5. Day 10: 97.1 ± 28.8
Villumsen et al. 2015 [39] (Denmark)	Changes in function assessed at admission and discharge by a physiotherapist using Timed Up and Go (TUG) and BI scores	TUG: Minority of participants (40.3%) performed better on discharge. BI Score: Majority of participants (73%) performed better on discharge

All figures mean ± standard deviation (SD) unless stated

*DEMMI* De Morton Mobility Index, *30sCST* 30 s Chair Stand Test, *BI* Barthel Index, *QF* Quadriceps force, *TUG* timed up and go

Adverse effects occurring during the period of monitoring were poorly reported, with only four studies reporting this outcome; two advised there were no adverse effects [34, 38] and two reported one death (unrelated to physical activity) [31, 32] during the course of their research.

### Sub-group analyses

Sub-group analyses were performed comparing studies at lower risk of bias (according to AXIS appraisal) and concerning only one medical condition to the overall physical activity and step count results. Both sub-group analyses found no significant difference in results comparing these devices to the overall results (Table 6), indicating the general results are an accurate representation of PA levels.

**Table 6:** Sub-group analyses

Group	Participants (*N*)	Results (Mean ± SD)	*p*-Value	95% Confidence interval
*All 24 hr PA studies*	413	6.6% ± 6.3		
24 hr PA Studies at lower risk of bias [35, 38]	65	7.8% ± 7.8	0.169	− 2.906 to 0.506
24 hr PA studies of Heart Failure patients [35]	27	7.3% ± 9.6	0.590	− 3.247 to 1.847
24 hr PA studies of COPD patients [32]	10	7.7% ± 5.6	0.585	− 5.043 to 2.843
*All step count studies*	1039	881.8 ± 1068.2		
Step count studies at lower risk of bias [35, 39, 40]	405	875.8 ± 1106.2	0.924	− 117.883 to 129.883
Step count studies of heart failure patients [33, 35]	72	705 ± 970.8	0.172	− 76.918 to 430.518

## Discussion

The aim of this review was to identify, evaluate and synthesise the evidence on the physical activity levels of acutely ill older patients undergoing treatment in an HaH vs inpatient setting. No HaH studies of older adults could be identified, representing a significant gap in the literature surrounding this treatment model. Despite the lack of HaH research in this field, this review has provided useful data on the baseline physical activity levels that could be expected for patients suitable for treatment in a HaH model of care: when monitored for 24 h/day, such patients spend on average 6.6% of the time active, and walk as few as 881.8 steps per day. These findings are consistent with other research on hospitalised older adults, despite the strict HaH-specific inclusion/exclusion criteria applied. Baldwin [14] reviewed 42 studies reporting the activity levels of acutely admitted medical and surgical adult patients, and found patients spent between 93% and 98.8% of their entire stay sitting or lying, and that the majority of studies reported a daily step count of < 1000. Similarly, Fazio [40], in a systematic review of standing/walking activity in medical inpatients, found that patients were active for 70 min per 24 h (4.9% of the time). The baseline PA values provided in this review may be suitable for use as an inpatient comparator value in future HaH PA studies.

The low levels of activity reflected in our findings can result in functional decline, however, in our results only four of the studies measuring physical activity also measured functional change. This represents a missed opportunity to further explore correlations between physical activity and functional decline that should be addressed in future PA studies in hospitalised and HaH patients. Where functional changes were reported there was high heterogeneity in results between studies. Agmon [41] established that walking less than 900 steps when hospitalised was strongly associated with functional decline in older adults. Both Ueda [31] and Villumsen [39] reported a mean step count below this threshold, and while both reported results using the Barthel Index, measurements were taken at different points in the studies and the results were presented differently: Ueda [31] reported the change in mean score, while Villumsen [39] reported the percentage of participants who improved. In all, six different metrics were used in the four studies reporting functional change, with high variability in measurement tools (see Online Resource 4), data collection protocols and reporting formats, precluding meaningful synthesis of the results. Assessing physical function in acutely ill older inpatients who may present with a wide range of medical conditions and functional levels is undoubtedly challenging, and research is ongoing to identify the most feasible tools to use in this patient group [42]. A consensus-driven core outcome set for studies of functional performance in either older or hospitalised populations has yet to be developed and should be a research priority to allow evaluation and meta-analysis of the findings of studies in this field.

Placing the findings of this review in the wider context of physical activity research is challenging again due to substantial differences in the methods and outcome measures used. The techniques most frequently utilised in the studies in this review (24 h recording, positional accelerometery) rarely feature in population or community-based research. Including night-time activity is likely to present a more accurate picture of all activity undertaken, especially in a hospital setting where circadian rhythms may be disrupted [14], but will result in lower average activity levels than studies of day-time PA or sedentary behaviour only. This is evident in the results for the three studies that conducted monitoring over a shorter, daytime, timeframe (Table 3) which found physical activity ranged from 8.8 to 13.9% of the monitoring period.

As a result of these different outcome measures, recording periods and a lack of objectively established normative values for the 24-h physical activity of healthy free-living older adults, it is challenging to establish how much activity drops when hospitalised. However, as the continuous objective monitoring of research participants becomes easier and cheaper with developments in accelerometery and wearable digital technology, it may be the case that normative values for PA in free-living older adults can be established. This would allow more accurate evaluation of the extent to which normal PA is impeded by acute illness, in both HaH and inpatient settings.

### Strengths and limitations of this review

A strength of this review is that it followed a systematic approach following Cochrane guidelines where applicable [20] and was reported in accordance with PRISMA statement, which reduces the risk of bias. A possible limitation of this review is its high specificity arising from highly refined inclusion and exclusion criteria. This led to some potentially relevant articles being excluded. For instance, two promising RCTs were identified during the literature search and selection process which found that adult HaH patients may around 2.6 times more active than inpatients [43, 44], however, these studies were excluded as it was not possible to isolate the results for participants aged over 60 years-only. A further limitation of this review is the high risk of bias present in the studies identified, which may limit the representativeness of the findings.

## Conclusion

Physical and functional decline, caused in part due to inactivity during hospital admission, can have a considerable impact on an older patient’s health and ability to remain independent on discharge. HaH may offer a treatment environment that preserves and facilitates physical activity in older patients, however, it has been demonstrated in this review that there is a lack of research evidence to confirm this. This review has provided an indication of the baseline activity levels of inpatients suitable for a Hospital at Home service, however primary objective research is needed in this treatment setting.

This review also identified that functional change is infrequently measured along with physical activity, representing a missed opportunity to assess the impact of immobility in hospital on function. Where they are reported, functional measures are highly diverse and data collection protocols vary, impeding comparisons between studies. A consensus-driven core outcome set for the investigation of functional decline in hospitalised patients would greatly facilitate the comparison and synthesis of research in this field.

### Changes to original protocol

Sedentary behaviour, defined as ‘any waking behaviour characterized by an energy expenditure ≤ 1.5 metabolic equivalents (METs), while in a sitting, reclining or lying posture’ [45], was included in the search strategy as a related field to physical activity. No studies reporting sedentary behaviour as the primary outcome met the inclusion criteria, therefore, this concept is not discussed further in this review.

## Electronic supplementary material

Below is the link to the electronic supplementary material.

Supplementary file1 (DOCX 20 kb)

Supplementary file2 (XLSX 28 kb)

Supplementary file3 (PDF 203 kb)

Supplementary file4 (DOCX 21 kb)
