# Supplementary material for: A systematic review of the physical activity levels of acutely ill older adults in Hospital At Home settings: an under-researched field
Source: Eur Geriatr Med. 2020 Oct 15;12(2):227–38. doi: 10.1007/s41999-020-00414-y (PMC7557152; doi:10.1007/s41999-020-00414-y)
Supplement: Supplementary file 1 — Supplementary file1 (DOCX 20 kb) [file 41999_2020_414_MOESM1_ESM.docx]

Online Resource 1: Search Strategies

**Title:** Physical activity in Acutely Ill Older Adults: Hospital At Home Vs Hospital Inpatient Care Settings – A Systematic Review

**Title:** Physical activity in Acutely Ill Older Adults: Hospital At Home Vs Hospital Inpatient Care Settings – A Systematic Review

**Authors:** Jennifer Scott, Ukachukwu O Abaraogu, Graham Ellis, Maria Giné-Garriga, Dawn A Skelton

**Corresponding Author:** Jennifer Scott, [jennifer.scott@gcu.ac.uk](mailto:jennifer.scott@gcu.ac.uk), ORCID <https://orcid.org/0000-0002-1481-4270>, Centre for Living, School of Health and Life Sciences, Glasgow Caledonian University, Glasgow, United Kingdom

**Search Strategies**

Filters

- All EBSCO Searches: English Language, abstracts available, humans, date 1980-present
- PEDro: 1980-present, within Gerontology subdiscipline
- Cochrane Library: Protocols, Trials and Reviews, 1980-present, term variants applied, searching titles abstracts and keywords
- OT Seeker: Gerontology

Key:

All EBSCO (Medline, Amed, CINAHL) Searches:

| **Search Operator:** | **Tag** |
| --- | --- |
| MeSH/Subject heading | MH |
| Abstract search | AB |
| Proximity operator in any order within ‘n’ words | N0, N1 etc. |
| Wildcard: any or no letter e.g. aging, ageing | # |
| Wildcard: truncation | * |

Cochrane Library/CENTRAL Searches:

| **Search Operator:** | **Tag** |
| --- | --- |
| Keyword search | Kw |
| Title search | Ti |
| Abstract search | ab |
| Proximity operator adjacent in any order | NEXT |
| Wildcard: any or no letter e.g. aging, ageing | ? |
| Wildcard: truncation | * |

Medline (EBSCO Interface):

| # | Query |
| --- | --- |
| S15 | S13 OR S14 |
| S14 | S9 AND S11 AND S12 |
| S13 | S9 AND S10 AND S12 |
| S12 | S7 OR S8 |
| S11 | S5 OR S6 |
| S10 | S3 OR S4 |
| S9 | S1 OR S2 |
| S8 | AB physical activit* OR sedentary N0 (behaviour or life#style* or time) OR Acceleromet* |
| S7 | MH Physical Functional Performance |
| S6 | AB In#patient OR "care of the elderly ward" OR "care of the elderly unit" OR (geriatric or medic* or acute) N0 ward OR (geriatric or medic* or acute ) N0 unit |
| S5 | MH Hospitalization or Inpatients |
| S4 | AB “Hospital at home” OR “Hospital in the home” OR “acute care at home” OR Admission N0 avoidance OR Hospital N0 outreach |
| S3 | MH Home Care Services, Hospital-based |
| S2 | AB Ag#ing OR Frail* OR Geriatric* OR “over 60 years” OR elderly OR older adult* |
| S1 | MH Aged OR Frail Elderly OR Geriatrics |

CINAHL (EBSCO Interface):

| # | Query |
| --- | --- |
| S15 | S13 OR S14 |
| S14 | S9 AND S11 AND S12 |
| S13 | S9 AND S10 AND S12 |
| S12 | S7 OR S8 |
| S11 | S5 OR S6 |
| S10 | S3 OR S4 |
| S9 | S1 OR S2 |
| S8 | AB In#patient OR "care of the elderly ward" OR "care of the elderly unit" OR (geriatric or medic* or acute) N0 ward OR (geriatric or medic* or acute) N0 unit |
| S7 | MH Hospitalization OR Inpatients |
| S6 | AB “Hospital at home” OR “Hospital in the home” OR “acute care at home” OR Admission N0 avoidance OR Hospital N0 outreach |
| S5 | MH Home Health Care |
| S4 | AB Ag#ing OR Frail* OR Geriatric* OR “over 60 years” OR elderly OR older adult* |
| S3 | MH Aged OR Aged Hospitalized OR Aged, 80 and Over OR Frail Elderly |
| S2 | AB In#patient OR "care of the elderly ward" OR "care of the elderly unit" OR (geriatric or medic* or acute) N0 ward OR (geriatric or medic* or acute) N0 unit |
| S1 | MH Hospitalization OR Inpatients |

AMED (EBSCO Interface):

Search String:

| # | Query |
| --- | --- |
| S6 | S1 AND S3 AND S4 |
| S5 | S1 AND S2 AND S4 |
| S4 | AB physical activit* OR sedentary N0 (behaviour or life#style* or time) OR Acceleromet* |
| S3 | AB In#patient OR "care of the elderly ward" OR "care of the elderly unit" OR (geriatric or medic* or acute) N0 ward OR (geriatric or medic* or acute) N0 unit |
| S2 | AB “Hospital at home” OR “Hospital in the home” OR “acute care at home” OR Admission N0 avoidance OR Hospital N0 outreach |
| S1 | AB Ag#ing OR Frail* OR Geriatric* OR “over 60 years” OR elderly OR older adult* |

PEDro:

Search String:

| Abstract and Title | Hospital at home physical activity |
| --- | --- |
| Subdiscipline | Gerontology |
| Published since | 1980 |
| Match Terms with | AND |
| Results: | 18 |

| Abstract and Title | Inpatient physical activity |
| --- | --- |
| Subdiscipline | Gerontology |
| Published since | 1980 |
| Match Terms with | AND |
| Results: | 12 |

Cochrane Library/CENTRAL:

(frail* OR Geriatric* OR "over 60 Years" OR elderly OR "Older adults") AND (inpatient OR in-patient OR (care of the elderly ward) OR (care of the elderly unit) OR ((geriatric* OR medic* OR acute) ward) OR ((geriatric* OR medic* OR acute) unit)) AND ((physical NEXT activit*) OR (physical NEXT function) OR (sedentary NEXT behaviour) OR (sedentary NEXT time) OR acceleromet*)

OR

(frail* OR Geriatric* OR "over 60 Years" OR elderly OR "Older Adults") AND ("hospital at home" OR "hospital in the home" or "acute care at home" OR (admission avoidance) OR (hospital NEXT outreach)) AND ((physical NEXT activit*) OR (sedentary NEXT behaviour) OR (sedentary NEXT time) OR acceleromet*)

Results: 10 Reviews, 610 Trials

OT Seeker:

| Line | Term |
| --- | --- |
|  | Hospital at home |
| OR | Inpatient |
| AND | Physical activity |
| Results: | 41 |
