# Supplementary material for: A systematic review of the physical activity levels of acutely ill older adults in Hospital At Home settings: an under-researched field
Source: Eur Geriatr Med. 2020 Oct 15;12(2):227–38. doi: 10.1007/s41999-020-00414-y (PMC7557152; doi:10.1007/s41999-020-00414-y)
Supplement: Supplementary file 3 — Supplementary file3 (PDF 203 kb) [file 41999_2020_414_MOESM3_ESM.pdf]

Online Resource 3: AXIS Appraisal

Title: Physical activity in Acutely Ill Older Adults: Hospital At Home Vs Hospital Inpatient Care Settings – A Systematic Review

Authors: Jennifer Scott, Ukachukwu O Abaraogu, Graham Ellis, Maria Giné-Garriga, Dawn A Skelton

Corresponding Author: Jennifer Scott, jennifer.scott@gcu.ac.uk, ORCID https://orcid.org/0000-0002-1481-4270, Centre for Living, School of Health and Life Sciences, Glasgow Caledonian University, Glasgow, United Kingdom

AXIS Quality and Risk of Bias Assessment

To facilitate the assessment risk of bias for each study as well as the literature included as a whole, the appraisal has been split into two sections: appraisal of quality of included studies (Table 4) and appraisal of the Risk of Bias across included studies (Table 5).

Table 1: AXIS Appraisal Results – Quality of Studies

Domain	Axis Question	Belala et al. 2019	Brown et al. 2009	Evenson et al. 2017	Fisher et al. 2016	Floegel et al. 2019	Karlsen et al. 2017	Lim et al., 2018	McCullagh et al. 2016	Ostir et al., 2013	Pedersen et al. 2013	Pitta et al., 2006	Rowlands et al. 2014	Salis et al., 2015	Ueda et al. 2016	Valkenet et al. 2019	Villumsen et al.
Reporting	Were the aims/objectives of the study clear?	+	+	+	+	+	+	+	+	+	+	+	+	+	+	+	+
Study Design	Was the study design appropriate for the stated aim(s)?	+	+	+	+	+	+	+	+	+	+	+	+	+	+	+	+
Study Design	Was the sample size justified?	-	-	-	-	-	+	-	-	-	-	+	-	-	+	-	+
Reporting	Was the target/reference population clearly defined?	+	+	+	+	+	+	+	+	+	+	+	+	+	+	?	+
Study Design	Was the sample frame taken from an appropriate population base so that it closely represented the target population under investigation?	+	+	+	+	+	+	+	+	+	+	+	+	+	+	+	+
Study Design	Were the risk factors and outcome variables measured appropriate to the aims of the study?	+	+	+	+	+	+	+	+	+	+	+	+	+	+	+	+
Reporting	Is it clear what was used to determine statistical significance and/or precision estimates?	+	+	+	+	+	+	+	+	+	+	+	+	+	+	+	+
Reporting	Were the methods (including statistical methods) sufficiently described to enable them to be repeated?	+	+	+	+	+	+	+	+	+	+	+	+	+	?	+	+
Reporting	Were the basic data adequately described?	+	+	+	+	+	+	+	?	+	+	+	+	+	+	+	+
Reporting	Were the results presented for all the analyses described in the methods?	+	+	+	+	+	+	+	+	+	+	+	+	+	+	+	+
Study Design	Were the authors discussions and conclusions justified by the results?	?	+	+	+	+	+	+	+	+	+	+	+	+	+	+	+
Reporting	Were the limitations of the study discussed?	+	?	+	+	+	+	+	+	+	+	+	+	+	+	+	+
Study Design	Were there any funding sources or conflicts of interest that may affect the author's interpretation of the results?	+	+	+	?	+	+	+	+	+	?	?	+	+	+	+	?
Study Design	Was ethical approval or consent of participants attained?	+	?	+	?	?	?	+	+	?	+	+	+	?	+	+	+

Key: + = Yes, - = No, ? = Unclear/Not reported

Table 2: AXIS Appraisal Results – Risk of Bias

Domain	Axis Question	Belala et al., 2019	Brown et al., 2009	Evenson et al., 2017	Fisher et al., 2016	Floegel et al., 2019	Karlsen et al., 2017	Lim et al., 2018	McCullagh et al., 2016	Ostir et al., 2013	Pedersen et al., 2013	Pitta et al., 2006	Rowlands et al., 2014	Salis et al., 2015	Ueda et al., 2016	Valkenet et al., 2019	Villumsen et al.
Selection	Was the selection process likely to select subjects/participants that were representative of the target population under investigation?	+	+	+	+	+	+	+	+	+	+	+	+	+	+	+	+
Selection	Were measures taken to address and categorise non responders?	-	-	+	-	+	?	-	-	-	-	-	-	-	-	-	-
Instrumentation	Were the risk factor and outcome variables measured correctly using instruments/measurements that had been trialled, piloted or published previously?	+	+	+	+	+	+	+	+	+	+	+	+	+	-	+	+
Selection	Does the response rate raise concerns about non-response bias (Response rate <80%, Yu and Tse, 2012)	+	-	+	-	-	-	-	+	+	-	-	-	-	-	-	-
Selection	If appropriate, was information about non-responders described?	+	-	+	-	+	-	-	-	-	-	-	-	-	-	-	-
Reporting	Were the results internally consistent?	+	+	+	+	?	+	+	+	+	?	+	+	+	+	+	+

Key: + = Low risk of bias, - = high risk of bias, ? = Some concerns
