# Supplementary material for: A systematic review of the physical activity levels of acutely ill older adults in Hospital At Home settings: an under-researched field
Source: Eur Geriatr Med. 2020 Oct 15;12(2):227–38. doi: 10.1007/s41999-020-00414-y (PMC7557152; doi:10.1007/s41999-020-00414-y)
Supplement: Supplementary file 4 — Supplementary file4 (DOCX 21 kb) [file 41999_2020_414_MOESM4_ESM.docx]

Online Resource 4: Range and Validity of Measurement Tools Used

**Title:** Physical activity in Acutely Ill Older Adults: Hospital At Home Vs Hospital Inpatient Care Settings – A Systematic Review

**Authors:** Jennifer Scott, Ukachukwu O Abaraogu, Graham Ellis, Maria Giné-Garriga, Dawn A Skelton

**Corresponding Author:** Jennifer Scott, [jennifer.scott@gcu.ac.uk](mailto:jennifer.scott@gcu.ac.uk), ORCID <https://orcid.org/0000-0002-1481-4270>, Centre for Living, School of Health and Life Sciences, Glasgow Caledonian University, Glasgow, United Kingdom

Range and Validity of Measurement Tools Used

| **Method/Instrument Used** | **Utilised in** | **Validity (Source of Reference)** |
| --- | --- | --- |
| 30 second Chair-Stand Test (30CST) | Karlsen et al., 2017 | Jones CJ, Rikli RE, Beam WC: A 30-s chair-stand test as a measure of lower body strength in community-residing older adults. Res Q Exerc Sport 1999;70:113–9 (Source: Karlsen et al., 2017) |
| ActivPAL (PAL Technologies, Glasgow, UK) | Evenson et al., 2017  Floegel et al., 2019  Karlsen et al., 2017  Rowlands et al., 2014  Villumsen et al., 2015 | Kozey-Keadle S, Libertine A, Lyden K, Staudenmayer J, Freedson PS. Validation of wearable monitors for assessing sedentary behavior. Med Sci Sports Exerc. 2011;43 (8):1561–1567. (Source: Floegel et al., 2019, Villumsen et al., 2015)  Bassett DR, John D, Conger SA, Rider BC, Passmore RM, Clark JM. Detection of lying down, sitting, standing, and stepping using two ActivPAL monitors. Med Sci Sports Exerc. 2014;46(10):2025–2029. (Source: Floegel et al., 2019)  Grant PM, Ryan CG, Tigbe WW, Granat MH. The validation of a novel activity monitor in the measurement of posture and motion during everyday activities. Brit J Sports Med. 2006;40:992–7. (Source: Rowlands et al., 2014, Villumsen et al., 2015)  Lyden K, Kozey Keadle SL, Staudenmayer JW, Freedson PS. Validity of two wearable monitors to estimate breaks from sedentary time. Med Sci Sports Exerc. 2012;44(11):2243–52. (Source: Rowlands et al., 2014) |
| Augmentech (Augmentech Inc, Pittsburgh, PA) | Brown et al., 2009  Pedersen et al., 2012 | Brown CJ, Roth DL, Allman RM. Validation of the use of wireless monitors to measure levels of mobility during hospitalization. J Rehabil Res Dev 2008;45: 551–558. (Source: Brown et al., 2009)  Also validated by Pedersen et al., 2013 as part of their published research study |
| Barthel index (BI) | Ueda et al., 2016  Villumsen et al., 2015 | Shah S, Vanclay F, & Cooper B. (1989). Improving the sensitivity of the Barthel index for stroke rehabilitation. *Journal of Clinical Epidemiology, 42*(8), 703–709. (Source: Villumsen et al., 2015)  Mahoney FI, Barthel DW. FUNCTIONAL EVALUATION: THE BARTHEL INDEX. Md State Med J. 1965; 14: 61–65. (Source: Ueda et al., 2016) |
| Behavioural Mapping | Belala et al., 2019  Valkenet et al., 2019 | No validity information provided in study  Kramer SF, Cumming T, Churilov L, et al., Measuring Activity Levels at an Acute Stroke Ward: Stroke Ward: Comparing Observations to a Device. BioMed Research International. 2013;2013:8.  Valkenet, K., Bor, P., Van Delft, L. & Veenhof, C., 2019. Measuring physical activity levels in hospitalized patients: a comparison between behavioural mapping and data from an accelerometer. Clinical Rehabilitation. 33(7), pp.1233-1240. Available from: 10.1177/0269215519836454. (Source: Valkenet et al., 2019) |
| de Morton Mobility Index (DEMMI) | Karlsen et al., 2017 | de Morton NA, Davidson M, Keating JL: The de Morton Mobility Index (DEMMI): an essential health index for an ageing world. Health Qual Life Outcomes 2008;6:63 (Source: Belala et al., 2019, Karlsen et al., 2017)  de Morton NA, Lane K: Validity and reliability of the de Morton Mobility Index in the subacute hospital setting in a geriatric evaluation and management population. J Rehabil Med 2010;42:956–61 (Source: Karlsen et al., 2017)  de Morton NA, Davidson M, Keating JL: Reliability of the de Morton Mobility Index(DEMMI) in an older acute medical population. Physiother Res Int 2011;16:159–69 (Source: Karlsen et al., 2017) |
| Dynaport MoveMonitor (McRoberts, The Hague, NL), | Pitta et al., 2006  Valkenet et al., 2019 | Pitta F, Troosters T, Spruit MA et al., “Activity Monitoring for Assessment of Physical Activities in Daily Life in Patients With Chronic Obstructive Pulmonary Disease.” *Archives of Physical Medicine and Rehabilitation* 86.10 (2005): 1979–1985. Web. (Source: Pitta et al., 2006)  de Groot S and Nieuwenhuizen MG. Validity and reli- ability of measuring activities, movement intensity and energy expenditure with the DynaPort MoveMonitor. Med Eng Phys 2013; 35(10): 1499–1505. (Source: Valkenet et al., 2019)  Fokkenrood HJ, Verhofstad N, van den Houten MM, et al., Physical activity monitoring in patients with peripheral arterial disease: validation of an activity monitor. Eur J Vasc Endovasc Surg 2014; 48: 194–200. (Source: Valkenet et al., 2019)  Valkenet K, Veenhof C. Validity of three accelerometers to investigate lying, sitting, standing and walking. PLoS One. 2019;14(5):e0217545. Published 2019 May 23. doi:10.1371/journal.pone.0217545 (Source: Valkenet et al., 2019) – Found to have an excellent sensitivity for sitting (94%), excellent PPV for lying and walking (100% and 99%), but a poor sensitivity (13%) and PPV (19%) for standing. |
| Functional Independence Measure (FIM) | Ueda et al., 2016 | Not reported in study  Validation found from general search: Dodds TA, Martin DP, Stolov WC, Deyo RA. A validation of the functional independence measurement and its performance among rehabilitation inpatients. Arch Phys Med Rehabil 1993;74:531–6. |
| Hand grip strength | Karlsen et al., 2017 | Roberts HC, Denison HJ, Martin HJ, et al: A review of the measurement of grip strength in clinical and epidemiological studies: towards a standardised approach. Age Ageing 2011;40:423–9 (Source: Karlsen et al., 2017) |
| Mediwalk, (Terumo, Japan) | Ueda et al., 2016 | Not Reported  No validation studies found in in general search |
| Quadriceps force (Cybex II dynamometer) | Pitta et al., 2006 | M Decramer, L.M Lacquet, R Fagard, P Rogiers, Corticosteroids contribute to muscle weakness in chronic airflow obstruction Am J Respir Crit Care Med, 150(1994), pp. 11-16 (Source: Pitta et al., 2006) |
| Stepwatch Activity Monitor (Modus health, Washington, US) | Ostir et al., 2013  Fisher et al., 2016  Lim et al., 2018  McCullagh et al., 2016 | Foster RC, Lanningham-Foster LM, Manohar C, et al., Precision and accuracy of an ankle-worn accelerometer-based pedometer in step counting and energy expenditure. Prev Med 2005;41:778-83. (Source: Fisher et al., 2016)  Resnick B, Nahm ES, Orwig D, Zimmerman SS, Magaziner J. Mea- surement of activity in older adults: reliability and validity of the Step Activity Monitor. J Nurs Meas 2001;9:275-90. (Source: Fisher et al., 2016)  Foster RC, Lanningham-Foster M et al (2005) Precision and accuracy of an ankle-worn accelerometer-based pedometer in step counting and energy expenditure. Prev Med 41:778–783 (Source: Lim et al., 2018, Ostir et al., 2013)  McCullagh, R., O'Connell, A.M., Dillon, C., Horgan, N.F. & Timmons, S. Comparative Accuracy of Motion Sensors for Frail-Older Hospitalised Patients. Irish Journal Of Medical Science, 2014 Galway. Irish Gerontological Society Conference, S354-S354. (Source: McCullagh et al., 2016)  Mudge, S., Stott, N. S. & Walt, S. E. 2007. Criterion validity of the StepWatch Activity Monitor as a measure of walking activity in patients after stroke. Arch Phys Med Rehabil, 88, 1710-5 (Source: McCullagh et al., 2016)  Cavanaugh JT, Coleman KL, Gaines JM, et al., Using step activity monitoring to characterize ambulatory activity in community-dwelling older adults. J Am Geriatr Soc. 2007; 55:120–124. (Source: Ostir et al., 2013) |
| Timed Up and Go (TUG) | Villumsen et al., 2015 | Podsiadlo, D., & Richardson, S. (1991). The timed “up & go”: A test of basic functional mobility for frail elderly persons. *Journal of the American Geriatrics Society, 39*(2), 142–148. (Source: Villumsen et al., 2015) |
| Tractivity (Kineteks Corp, Canada) | Floegel et al., 2019 | Validated in Sallis R, Roddy‐Sturm Y, Chijioke E, Litman K, Kanter MH, Huang BZ, Shen E, Nguyen HQ. Stepping toward discharge: level of ambulation in hospitalized patients. J Hosp Med. 2015; <https://doi.org/10.1002/Jhm.2343>. |
